# Long-term outcome for patients with arthrogryposis multiplex congenita

**DOI:** 10.1007/s11832-015-0692-6

**Published:** 2015-10-26

**Authors:** Jean Dubousset, Michel Guillaumat

**Affiliations:** 23bis Rue Des Cordelières, 75013 Paris, France; Orthopedic department, Hopital Saint Joseph, Rue Raymond Losserand, 75014 Paris, France

**Keywords:** Arthrgryposis, Adult quality of life, Indications for treatment

## Abstract

**Purpose:**

To access the long-term outcomes for patients with arthrogryposis multiplex congenita at adult age.

**Materials and Methods:**

The cases were traced for most of them thanks to direct contact maintained from child hood, from colleagues interested from other parts of the country, and from the list from Alliance arthrogryposis association (parents and patients). The methods used were: mostly direct clinical examination, some phone calls or email. All answered a questionnaire for general life and mainly for function. One of these questions was: what is the function you missed most during your life?

**Results:**

65 patients( 41 females and 24 males) were reviewed at adult age from 22 to 65 years. For the personal life: 38 are married and had 34 children with only 4 having arthrogryposis. Only 27 (15 F/12 M) were living alone. Self-sufficiency was observed in 35, partial in 20, total dependence in 10 people. 38 reached university level, 20 had secondary school level, 10 had primary school level. Ambulation was made possible with wheelchair: permanent for 18, partial for 9 ambulating at home only, 8 ambulating outside with crutches and 29 were free walkers. 36 patients out of 65 were driving a car sometimes modified with special equipment. The involvement of spine was seen in 26 patients requiring surgical treatment sometimes complex combining anterior and posterior approach in 14 cases. Surgery of the lower limbs (hips, knee, feet) was very often repeated, with almost always stability, pain free and function. The most important finding was that 52 patients had more or less severe involvement of the upper limbs which was considered by the majority of the patients to be the most disabling, more than the absence of walking!

**Conclusion:**

Finally, it appeared that for the care of these patients, priority goes to the upper limbs function, because majority of these patients have a high level of intelligence. A remarkable fact is that many of these patients had to spend a lot of time during infancy and childhood in rehabilitation centers with education adapted for schools and teachers. Finally, they are grateful for that, telling often that it would have been much more difficult if not impossible to have such a treatment and education at home.

## Introduction

There are few long-term studies of how children with arthrogryposis function as adults. We have previously published reviews of children with arthrogryposis seen in the orthopaedic department of St Vincent de Paul & Saint Joseph Hospitals, Paris [1, 3] and in Lyon, France [2].

For the EPOS Symposium 2012, a longer follow-up of these and additional French adults with arthrogryposis was undertaken.

## Materials and methods

Adults with arthrogryposis were traced using the following contacts:

1. Children previously reported where contact had been maintained.
2. Details provided from other French orthopaedic surgeons with similar interests.
3. Contact details provided by the patients’ and parents’ association (Association Alliance Arthrogrypose), located in Grenoble, France.

Details of their condition were then obtained either by direct contact with clinical examination or by phone or email using a questionnaire.

## Questionnaire used

The following details were requested via a questionnaire:

1. Name, age, contact details
2. Details of arthrogryposis
  - Limb involvement
  - Spine invovement
3. Type of arthrogryposis if known
4. Treatment undertaken
5. Educational achievement
6. Profession
7. Mobility status
8. Activities of daily living
9. Social status

In addition all were asked which function or functions they most missed in adulthood and to provide any other comments. If possible a digital photograph was requested.

## Results

A total of 65 adults with arthrogryposis were reviewed. Their details are shown in Table 1.

**Table 1:** Patient details

Age (years)	Total	Female	Male
20–30	19	13	6
30–40	24	17	7
40–50	11	6	5
50–68	11	5	6

The social status of those reviewed is shown in Table 2.

**Table 2:** Social status

Social status	Total	Female	Male
Single	27	15	12
With partner	38	26	12

**Fig. 1:**
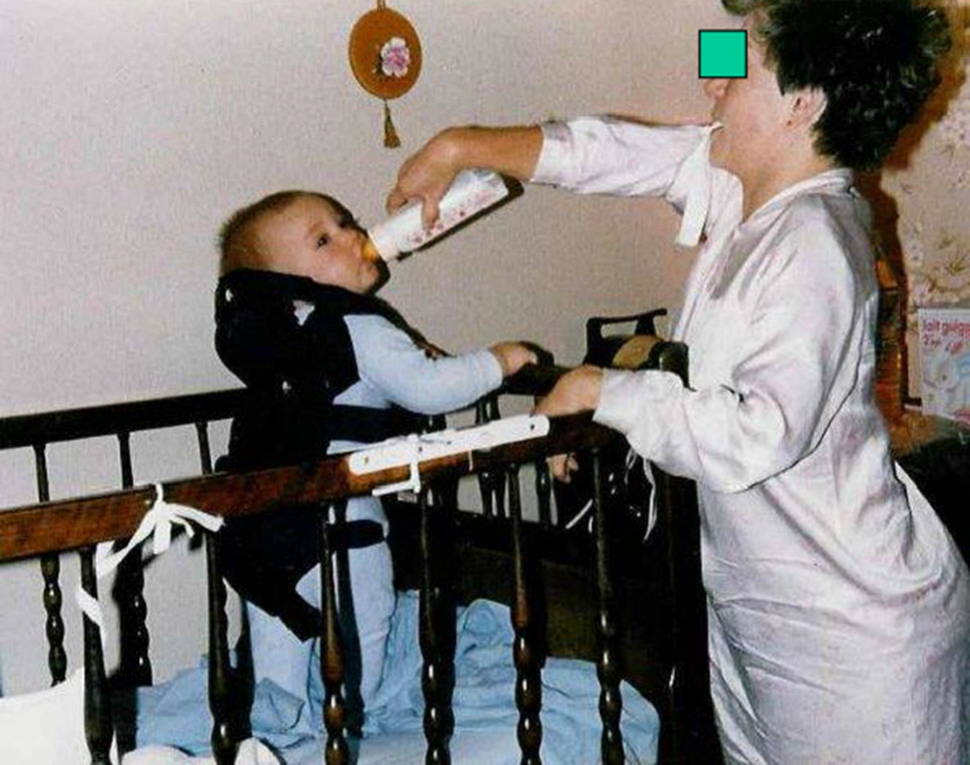
65 patients with arthrogryposis multiplex congenita (AMC) reviewed; 38 couples with 34 children; only 4 with AMC; all parents with AMC distal type

A total of 34 children had been born to the couples and of these 4 had arthrogryposis (Fig. 1). All were from parents who had the autosomal dominant form of distal arthrogryposis.

The type of arthrogryposis was difficult to assess, as of those contacted by questionnaire 32 replied “unknown”, 26 responded “amyoplasia” and 5 “distal type”. Two responded “congenital myopathy” but this diagnosis was not conclusive despite a muscle biopsy.

The limb involvement is detailed in Table 3.

**Table 3:** Limb involvement

Involvement	Number
Upper and lower limbs and spine	26
Upper and lower limbs	19
Upper limbs only	7
Lower limbs only	9
Distal type	5

A total of 26 had spinal involvement. This is a larger proportion than previously reported in the literature but is probably a consequence of the surgical interests of the authors.

All but one of these patients had had surgery during childhood and most of them had had repeated procedures to address the changes occurring with growth. The surgery was done on feet, knees, hips and spine, as well as upper limbs, shoulders, elbows, wrist and hands. Very few patients claimed to remember pain or disability secondary to the surgery. Only one patient, who had undergone a triceps transfer in front of the right elbow to give him some active flexion but who subsequently developed a flexion contracture of this elbow, claimed that he had not been sufficiently informed about the risks of this surgery at age 10. He said that if he had had the choice at the age of 10 he would have refused!

The level of independence and mobility is shown in Table 4.

**Table 4:** Independence and mobility

Status	Number	
Completely dependent on carer	10	
Partially dependent for washing and dressing	20	
Completely independent	35	
Mobility		Car driver
Independent mobility, no splints, modified shoes only	30	22
Crutch walking +/− splints	8	5
Indoor walking, wheelchair outdoors	9	
Permanent wheelchair	18	9

One respondent replied that being dependent for toileting was the worst handicap and a “misery”. Those who were independent had learnt coping strategies, such as using the mouth for holding tools.

A folding wheelchair was favoured by those with good upper limb function, particularly if able to drive, so as to be able to put the chair in the car.

Of the 64 reviewed, 36 were able to drive an adapted car; 13 of these classified themselves as partially dependent and 23 totally independent.

**Fig. 2:**
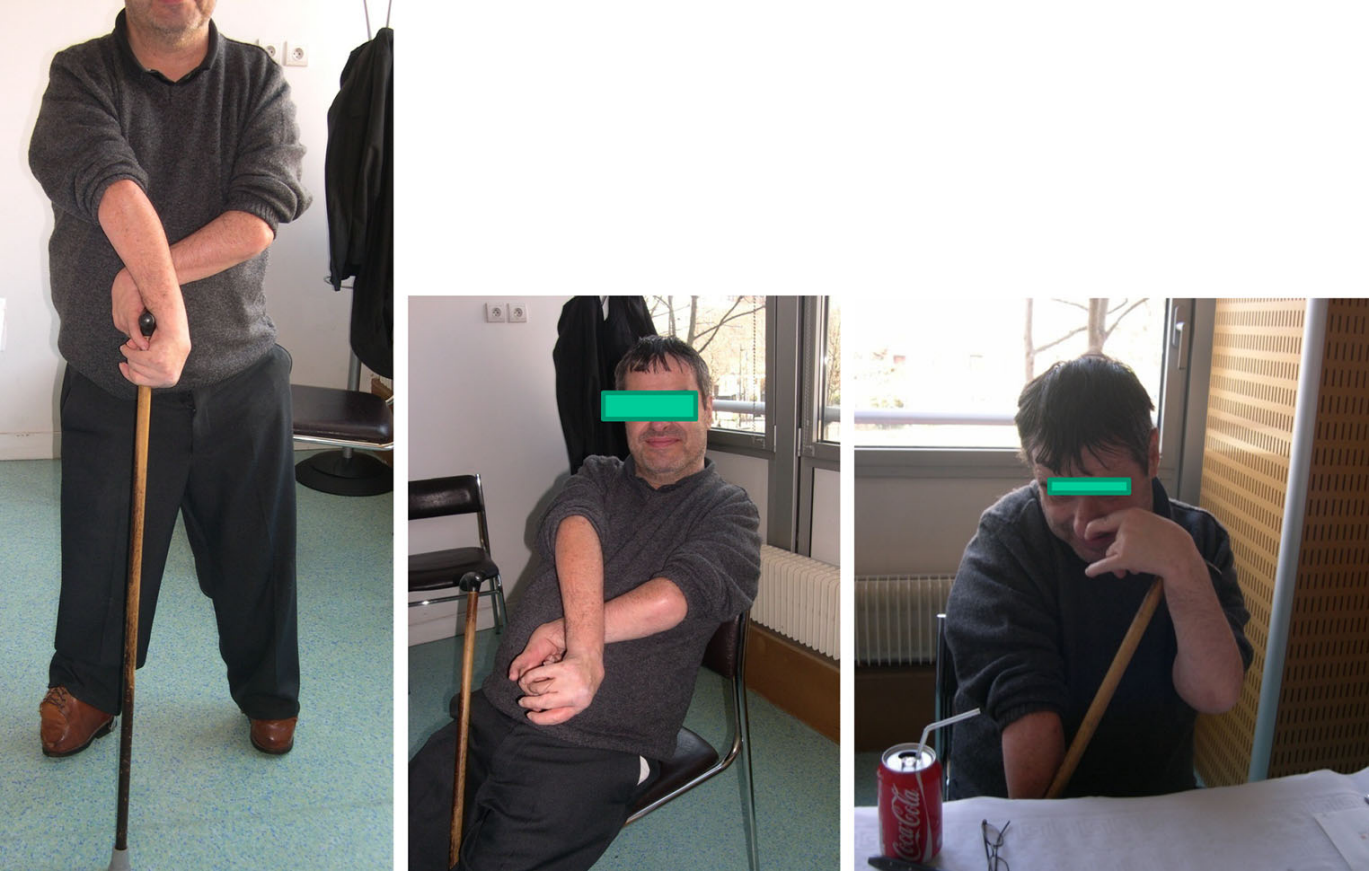
JMB. AMC 4 limbs. 54-year-old computer engineer with 3 children. Independent walker with stick and lower limb braces. Drives car. Only passive motion of the left elbow; “it is so good to touch the nose!”

An example of a man with independence despite four-limb involvement is shown in Fig. 2. This 57-year-old male is married with 3 children. He is freely ambulant with splints and crutches. As a child he had had multiple surgical procedures, including a posterior release of the left elbow. Despite enabling passive flexion only, he reported that it was “good to be able to scratch my nose!” He is working full-time as a computer engineer and drives his own car about 50,000 km per annum.

The educational achievements and subsequent employment are detailed in Table 5.

**Table 5:** Education and employment

Educational achievenment	Number
Attended local school	22
Attended specialised institution	33 (mainly with total body involvement) (10 failed to respond)
Achieved elementary grade	14
Achieved secondary school level	20
Attended university	31
Employment	
Unemployed	10
Student at university	5
Employed	38
Self-employed	11

Those in employment were in a variety of occupations with 38 in administrative positions. Among the 11 who were self-employed were painters, musicians, actors, lawyers, a farmer, a pharmacist and a physician.

### Functional deficit

All adults were asked “what is the function you most miss during your adult life?”

The replies are shown in Table 6.

**Table 6:** Functional deficit

Activity	Number
Being dependent for washing and bathroom	10
Impaired upper limb function	21
Unable to ride bicycle	15
Unable to drive car	6

One commented that inability to manage his toileting made his life a misery and another lamented not being able to catch his daughter in his arms.

## Regional involvement

### Spine

26 of the 64 adults had involvement of the spine. This represents 40 %, which is higher than the 25–30 % usually quoted. This is probably a reflection of the authors’ interest in spinal deformity. 14 patients had a curve of sufficient magnitude to require an instrumented fusion. Casts and braces were usually found to be ineffective when initiated for clinically evident deformity. In a few cases, night bracing was effective in preventing progression of a minor deformity, enabling achievement of skeletal maturity with a minor curve with no functional deficit.

The deformities were scoliosis and lordosis with severe stiffness.

We recommend that night bracing be commenced as soon as any deformity with axial rotation is detected. Spinal surgery can be performed from early childhood onwards, for example to enable a suitable erect posture for wheelchair use in severe cases. Techniques used have involved anterior, posterior and combined approaches.

An example is a severely affected girl with total body involvement born with a lordosis such that her head was touching her buttocks. Initial treatment was with traction and serial casting which enabled her to lie flat but with a significant lateral curve. At age 10 an anterior thoraco-lumbar fusion was performed with Dwyer instrumentation. Two years later the fusion required extending because of imbalance of the shoulders and excessive thoracic lordosis. A posterior fusion using Harrington instrumentation was undertaken. As she had sufficient hip flexion she was subsequently able to sit well in an electric wheelchair which she was able to control with a mouth-held joystick operated by moving her head (hand involvement prevented her using her upper limbs). She is currently 45 years old, living in an institution.

In patients who are still ambulant, a thoracic deformity may be associated with a severe thoraco-lumbar lordosis and respiratory compromise. A fusion may have to extend to the lumbar spine and then causes difficulties with walking. Surgery should be undertaken, however, as soon as respiratory function or walking becomes impaired.

In patients ambulating with splints who at puberty develop a progressive thoraco-lumbar scoliosis, an anterior instrumented fusion can be performed. Modern fixation techniques enable mobilisation without external splintage and a quick recovery of normal ambulation.

**Fig. 3:**
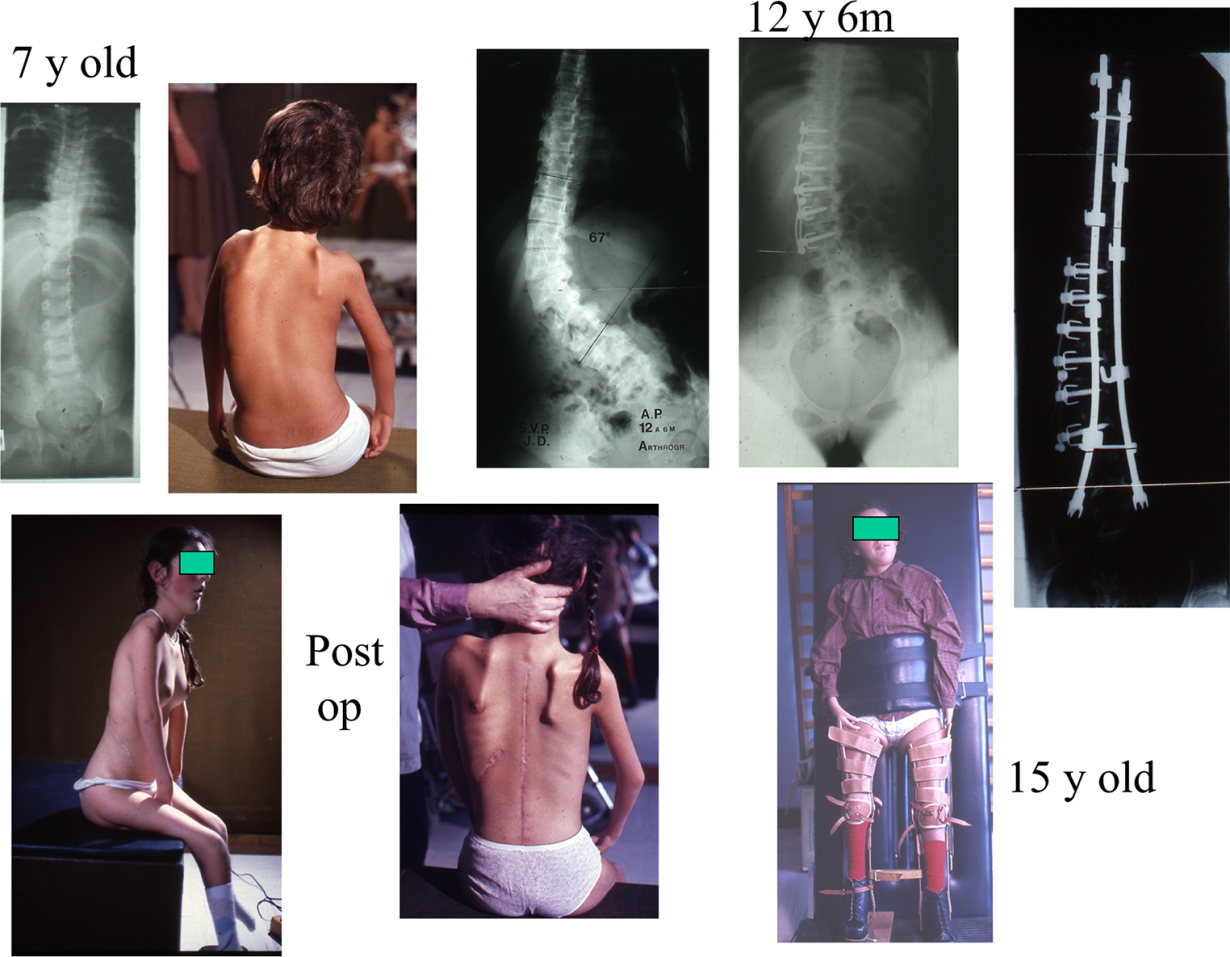
AMC 4 limbs and trunk

**Fig. 4:**
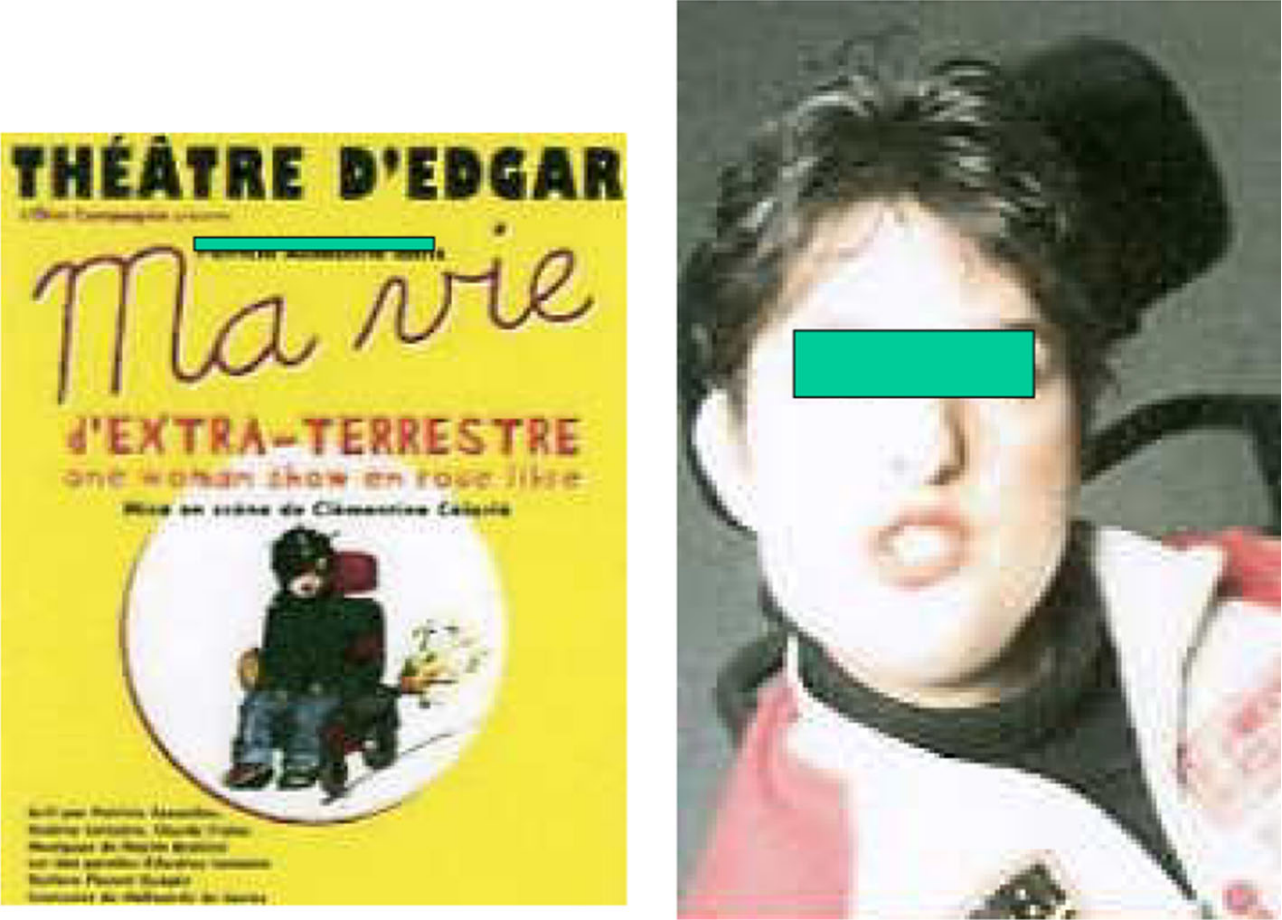
At review, 41-year-old theatre actress in wheelchair (illustrated at age 36)

**Fig. 5:**
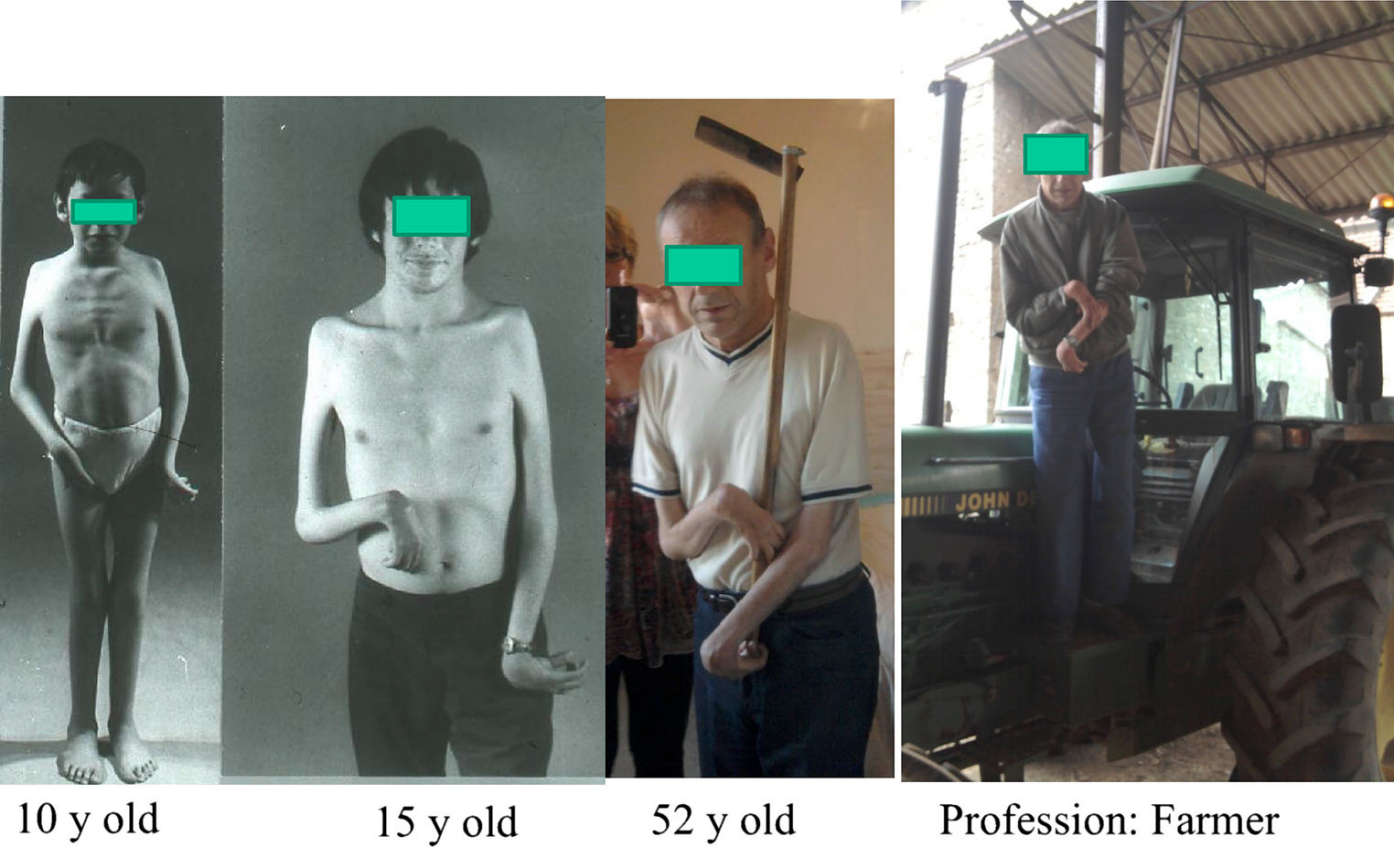
AMC, upper limbs predominant, transfer triceps right side at age 10 years. Progressive flexion contracture with loss of extension and bad functional result

In non-ambulant patients with pelvic obliquity, the fusion may need to be extended to the pelvis in order to correct sitting balance. An example is shown in Figs. 3 and 4. This girl required an anterior fusion followed by a posterior fusion with Cotrel–Dubousset (CD) instrumentation at age 15 because of a severe lumbar deformity. This enabled very satisfactory function in her wheelchair and at age 35 she became an actress in her wheelchair in a famous show in Paris. She is now 41 and maintains very good stability (Fig. 5).

### Upper limbs

The functional prognosis depends on finger function. Careful assessment is essential. A flexed wrist with good finger function is preferable to a wrist fused in extension with stiff fingers. If a child functions well holding a pen or pencil with both hands, one supine and the other prone, do not sacrifice this.

A patient with rigid fingers but a thumb with good opposition can function remarkably well. One of the patients in this series is now 35 with severe four-limb and trunk involvement. He has very little movement of his left hand, which is almost completely supinated, but has some grip between thumb and forefinger. He is wheelchair-dependent but is able to control his wheelchair using a joystick with his left hand. He is independent in the bathroom as he is able to roll from bed to chair and to toilet. He lives in Lille and travels on the TGV (the high-speed train) to Paris every day where he works as a computer expert.

Frequently the upper limbs are internally rotated at the shoulders with the elbows in extension but with good finger function. Early and frequent manipulations are essential to try and maximise movement. The aim is to achieve one hand coming to the mouth for feeding and one to the perineum for toileting. A derotational humeral osteotomy may be beneficial to place the hands in a functional position, combined with a posterior release of the elbows to achieve a passive range of flexion to 90°. Although only passive elbow flexion may be achieved, the patients learn to bring the hand to the mouth by resting the forearm on a stable object, e.g. the edge of a table. One patient in this series, a 45-year-old mother of a 10-year-old, uses a rubber band around her forearm to manipulate her upper limb by gripping the rubber band in her mouth and teeth, so enabling her to work full-time as a secretary.

One such patient in this series with four-limb involvement underwent bilateral humeral derotation osteotomies and posterior elbow releases, in addition to lower limb surgery, and at age 45 continues to be ambulant and is able to work as a sculptor of wood and stone because of the quality of her finger function.

With a mobile elbow without active flexion (either spontaneously or following a release), a muscle transfer can be performed. It is essential to carefully assess the child: the older the age at surgery the greater the risk of losing compensatory trick movements. A latissimus dorsi transfer is possible but may compromise crutch or stick use. In addition, following a transfer to achieve flexion, a progressive flexion contracture may be observed, compromising an initially satisfactory result. This may necessitate an extension osteotomy of the humerus to adjust the range of motion. Do not do a complete triceps transfer: we have seen a patient in whom, following a transfer at age 10, a severe flexion contracture had developed by the end of growth such that the patient wished it had not been performed.

**Fig. 6:**
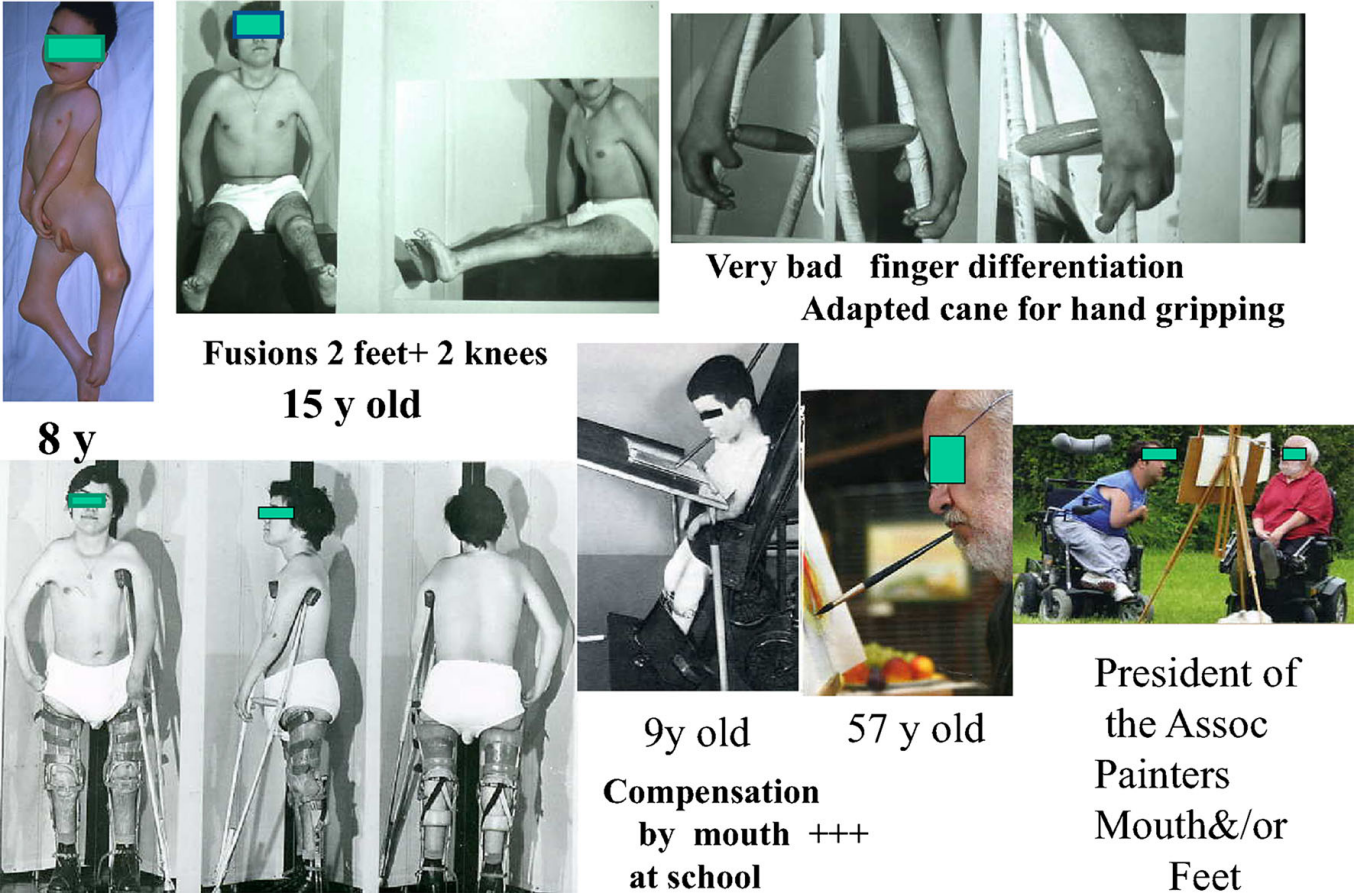
AMC 4 limbs. Differentiation of the fingers and even a little motion is essential

Patients often rely on their mouths to compensate for loss of upper limb function; one adult commented that he uses his mouth for opening and closing windows and the door of his freezer. Three patients in this series are artist painters with their mouths and one of them became the President of the International Association of the Painters by Mouth and/or Feet (Fig. 6).

## Lower limbs

### The hip

**Fig. 7:**
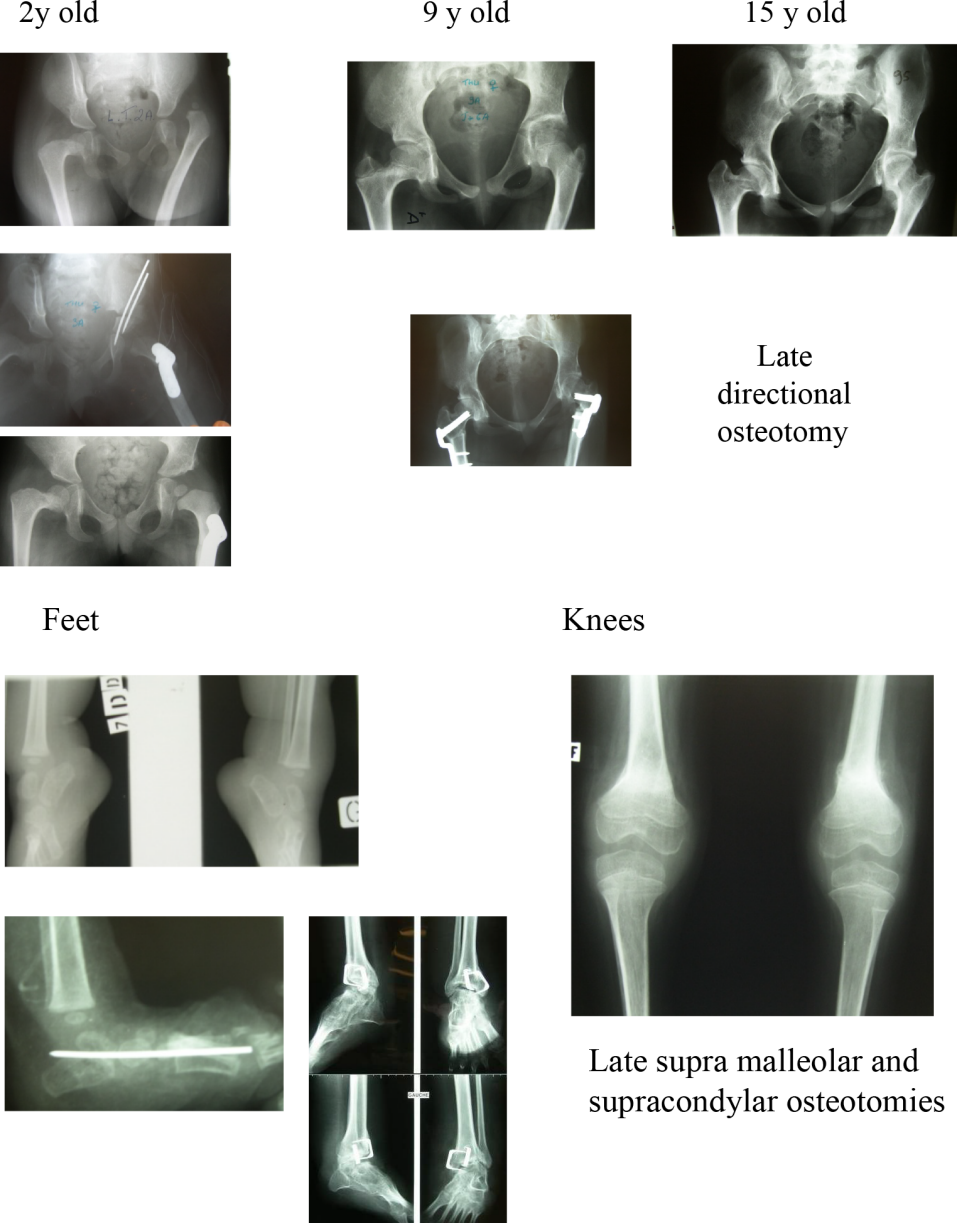
AMC 4 limbs. Now 31 years old. Home free walker, outside wheelchair. Occupation, full time switchboard operator

From the adult perspective, impairment of lower limb function was judged less disabling than upper limb. Walking is useful but can be compensated for by use of a wheelchair (Fig. 7).

Unilateral dislocations are generally treated by open reduction before 1 year of age. Caution is required with bilateral dislocations, particularly with stiff knees and feet, in case a stiff hip is generated. One patient in this series underwent repeated open reduction procedures for bilateral dislocations but by age 9 had bilateral stiff hips which were salvaged by bilateral proximal femoral resections, enabling comfortable sitting.

**Fig. 8:**
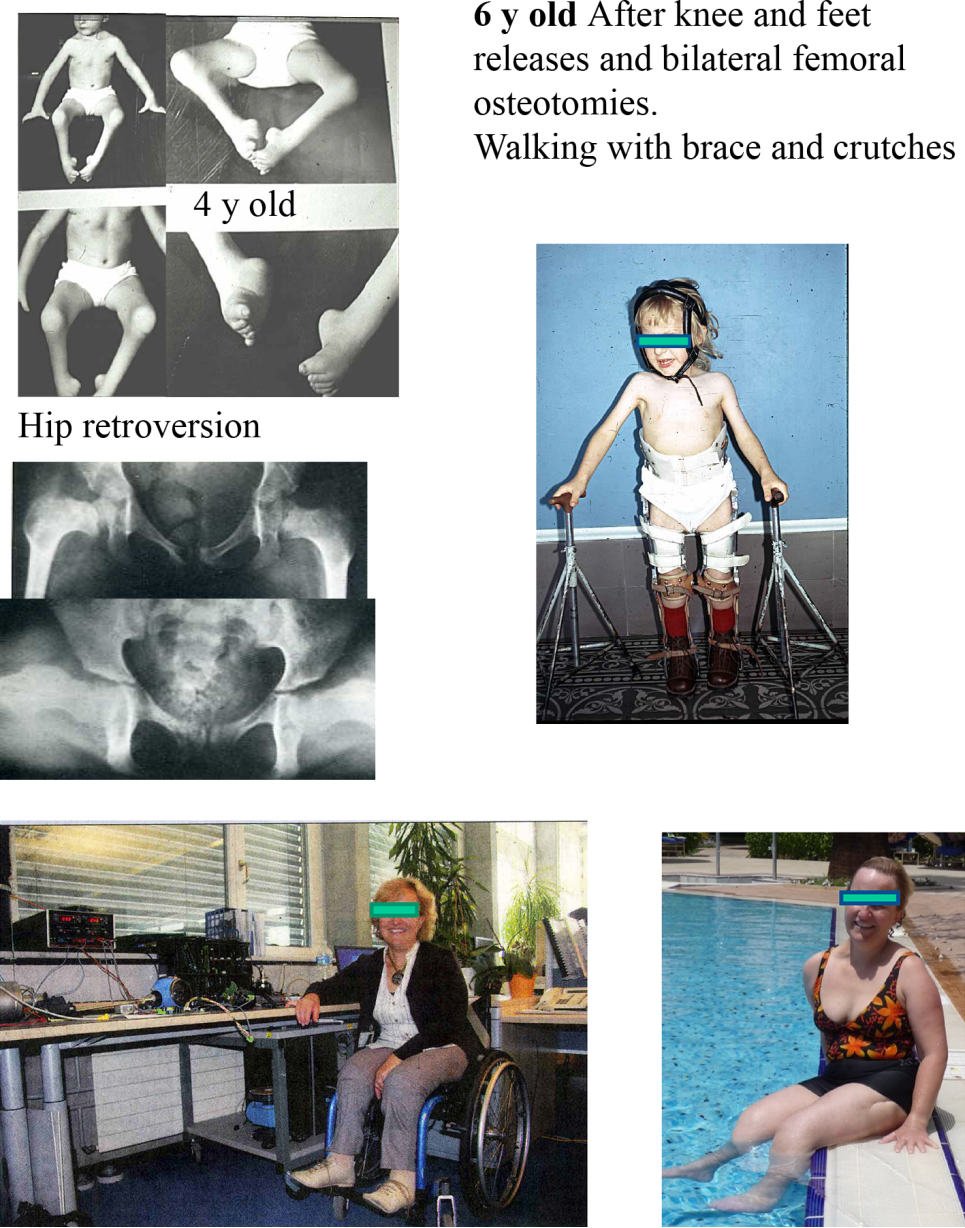
AMC lower limbs. 48 years old, married 2 children profession: Engineer Chief of Projects +/− Wheelchair permanent

The other problem observed in this series was severe bilateral hip flexion contractures without dislocation but with femoral neck retroversion. Despite derotation osteotomies and anterior releases, full extension was difficult to achieve and lumbar spine hyperlordosis, sometimes painful, was required for walking (Fig. 8).

### The knee

Flexion or hyperextension contractures responded to surgical releases, sometimes repeated. Little disability was reported although 15 patients reported that inability to cycle was their main concern.

### The feet

**Fig. 9:**
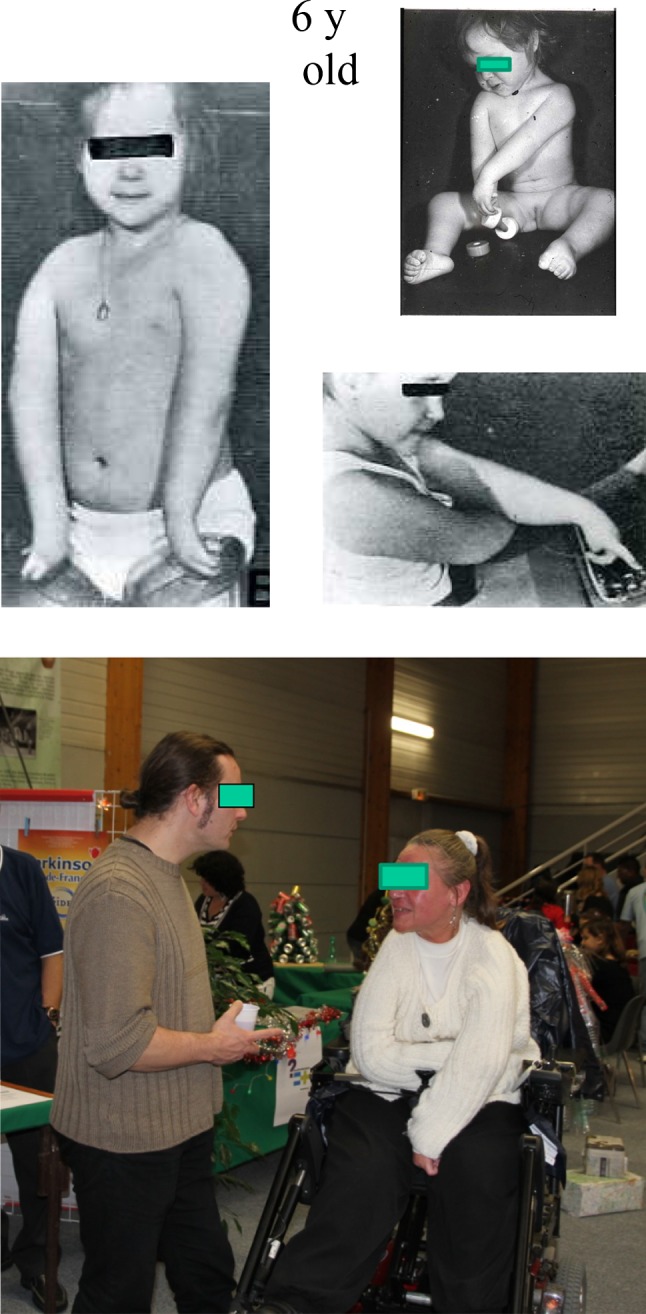
AMC 4-limb involvement. Stiffness and weakness, bad finger differentiation, R humeral derotation osteotomy, + R elbow posterior release. 47 years old, Masters degree in law. Full time Town Councillor for town planning, communication, transportation and architecture

The majority of patients reported that stiffness was not a problem for either walkers or non-walkers, provided the orientation was satisfactory. Problems were reported with trophic changes or chronic regional pain following multiple procedures (Fig. 9). A realignment supramalleolar osteotomy near skeletal maturity may be preferable to repeated foot procedures during growth resulting in increasing stiffness.

### Obesity

In 3 of the non-ambulant patients severe obesity was a problem and interfered with transfers, washing and dressing. In addition it made the work of any carer more difficult.

## Conclusions

1. Most children with arthrogryposis are of normal intelligence and continued schooling during any surgery or rehabilitation is essential.
2. Priority should be given to the upper limbs. The goal should be “one hand to the perineum and the other to the face, mouth, nose … (hair?)”.
3. Start with physiotherapy stretching. Consider humeral osteotomy for shoulder malalignment. Be careful with muscle transfers to achieve elbow flexion because of the risk of increasing elbow flexion contracture.
4. A flexed wrist with active finger flexion is preferable to an extended and stiff wrist with less finger movement.
5. Be careful with surgery in the older child who has learnt compensatory movements, where there is the risk of losing function.
6. Following trauma, try and restore the pre-trauma alignment, even if not normal, because even a 10° alteration in alignment may be a disaster for function.
7. Being upright is good in young children but do not wait too long to train those who will be non-ambulant to use a wheelchair.
8. Reduce unilateral dislocated hips but be cautious with bilateral dislocations.
9. Be careful to prevent soft tissue damage with foot surgery to prevent post-operative trophic changes.
10. The management of spinal deformities is similar to that in patients with muscular dystrophy, but with the advantage that arthrogryposis multiplex congenita is not a progressive disease. Surgical approaches can be anterior, posterior or combined. The aim should be to achieve the best three-dimensional correction. Appropriate anaesthetic and intensive care facilities are essential.
11. This study, with follow-up to age 68 (the oldest patient reviewed), has shown that there is no significant loss of function beyond adolescence.

## References

[1] Pous JG et al (1981) l’Arthrogrypose pendant l’enfance, arthrogryposis multiplex congenita. Chir Pediatr 22:289–364 7285264

[2] Fassier A, Wicart P, Dubousset J, Seringe R (2009) Arthrogryposis multiplex congenita. Long-term follow-up from birth until skeletal maturity. J Child Orthop 3(5):383–390 10.1007/s11832-009-0187-4 PMC2758174 19669823

[3] Dubousset J (2009) Arthrogrypose. Encycl Med Chir Paris App Locomoteur 15201 A 10

